# LFB: A Novel Antimicrobial Brevinin-Like Peptide from the Skin Secretion of the Fujian Large Headed Frog, *Limnonectes fujianensi*

**DOI:** 10.3390/biom9060242

**Published:** 2019-06-21

**Authors:** Bin Li, Peng Lyu, Shuping Xie, Haixin Qin, Wenyuan Pu, Houxi Xu, Tianbao Chen, Chris Shaw, Lilin Ge, Hang Fai Kwok

**Affiliations:** 1 Jiangsu Key Laboratory for Functional Substance of Chinese Medicine, Nanjing University of Chinese Medicine, 138 Xianlin Avenue, Qixia District, Nanjing 210000, China; bli0503@163.com (B.L.); shupingxie@outlook.com (S.X.); haixin0831@126.com (H.Q.); wenyuanpu@foxmail.com (W.P.); houxixu@126.com (H.X.); 2 Cancer Centre, Faculty of Health Sciences, University of Macau, Avenida de Universidade, Taipa, Macau SAR, China; penglyu00@163.com; 3 College of Biological Science and Technology, Fuzhou University, Fuzhou 350108, China; 4 Natural Peptide Discovery Group, School of Pharmacy, Queen’s University Belfast, Belfast BT9 7BL, Northern Ireland, UK; t.chen@qub.ac.uk (T.C.); chris.shaw@qub.ac.uk (C.S.)

**Keywords:** amphibian, Brevinin-like peptide, antimicrobial peptides, anticancer peptide, drug discovery

## Abstract

Amphibians are a natural source of abundant antimicrobial peptides and thus have been widely investigated for isolation of such biomolecules. Many new antimicrobial peptide families have been discovered from amphibians. In this study, a novel antimicrobial peptide named *Limnonectes fujianensis* Brevinvin (LFB) has been identified in the skin secretion from the Fujian large headed frog, *Limnonectes fujianensis*. The cDNA sequence was cloned from a skin secretion library and the predicted mature peptide was identified through MS/MS fragmentation sequencing of reverse phase HPLC fractions on the same sample. LFB was predicted to be an amphipathic, hydrophobic, alpha helical, and beta turn peptide that inserts into a lipid bilayer in order to kill the cells. In antimicrobial assays, a synthetic replicate of this novel antimicrobial peptide demonstrated significant activity against the Gram-positive bacterium *Staphylococcus aureus*, the Gram-negative bacterium *Escherichia coli* and the yeast, *Candida albicans*. This novel peptide was highly potent (MIC 4.88 uM) against Gram-negative bacterium, and also has the ability to inhibit the growth of human cancer cell lines with IC_50_ values ranging from 18.9 μM down to 2.0 μM. These findings help to enrich our understanding of Brevinin-like peptides. Moreover, the data presented here validate frog secretion as a source of potential novel antimicrobial peptides, that also exhibit anti-tumor properties, that could be useful for the treatment of cancer.

## 1. Introduction

In nature, frog’s skin has an important function to act as a crucial chemical and physical barrier to microbes. Follow any injury and/or sympathetic nervous stimulation, within the dermal granular glands of the frog’s skin antimicrobial peptides (AMPs) are synthesized, stored and secreted [1,2,3]; they are then able to act as the effector biomolecules of the innate immune system. In general, AMPs are relatively small (approximately ten to fifty amino acids), positively charged and amphipathic peptides which bind preferentially to microbial membranes [4]. Nowadays, most of the amphibian species studied produce many types of AMPs which can act alone or in synergy with other synthetic chemical drugs to inhibit a range of microbes [5,6,7,8].

Magainins were the first well-known AMPs isolated from an amphibian skin of the African clawed frog, *Xenopus laevis* [9]. According to the study, it was found that numerous AMPs are present among many species of amphibians, presumably for defense against pathogenic organisms [10,11]. The broad-spectrum antimicrobial activities can be chemically synthesized readily; these peptides have become the focus of significant research, spanning some 30 years now [12,13,14,15]. Due to their diversities of primary and secondary structural characters, AMPs from this source have been classified into several specific, structurally-related peptide families. These include the brevinins, esculentins and temporins in *Rana* species [16,17]. Despite having positive broad-spectrum activities, the hemolytic activities of AMPs limit their potential therapeutic applications in patients [18].

Brevinins are one of the most ubiquitous antibacterial peptides, which have two subfamilies: Brevinin-1 (with 24 amino acid residues) and Brevinin-2 (with 33 amino acid residues) [19,20,21,22]. These Brevinin-1 and -2 peptides demonstrated antimicrobial activities and enriched in the skin secretion of *L. fujianensis.* The broad antimicrobial activity and potential anti-cancer ability of Brevinin caused our interest in investigating its molecular mechanism using one of the most common frogs in South-East China, the Fujian large-headed frog, *L. fujianensis* [23]. Here, we report a novel antimicrobial peptide named *Limnonectes fujianensis* Brevinvin (LFB) which has been identified from the skin secretion of Fujian large-headed frog, *L. fujianensis*. LFB belongs to the Brevinin family and its structural conformation reflects the presence of three basic characteristic physico-chemical attributes: α-helix, β-sheet and random coil. LFB displayed notable potency against the Gram positive bacterium *S. aureus*, the Gram negative bacterium *E*. *coli* and the yeast *C*. *albicans*. Furthermore, LFB could also inhibit the growth of human cancer cell lines, with IC_50_ values ranging from 18.9 μM down to 2.0 μM.

## 2. Materials and Methods

### 2.1. Acquisition of Skin Secretions

Skin secretions were harvested from specimens of *L*. *fujianensis* (ten of the Fujian large headed frogs were applied) by electrical stimulation (4 ms pulse width, 50 Hz, 6 V) in accordance with the method of Tyler et al. [24], and the skin secretions were carefully washed from the skin using double deionized water, then snap-frozen in liquid nitrogen for lyophilization. Finally, the freeze-dried samples were then stored at −20 °C prior to analysis. This study is approval by the Nanjing University of Chinese Medicine Ethical Review Board (Approval Code: SYXK (SU) 2018-0048).

### 2.2. Encoding Novel Peptide Biosynthetic Precursors–Shotgun cDNAs Cloning

The cell mRNA protection buffer (Dynal Biotech, Wirral, UK)/cell lysis (1ML) was employed to dissolve 5 mg of lyophilized *L. fujianensis* skin secretion sample, and magnetic Dynabeads oligo (dT)_25_ (Dynal Biotech, UK) were used to isolate the polyadenylated mRNA, as described in supplier’s instructions. The isolated polyadenylated mRNA was then subjected to 5′ and 3′-rapid amplification of cDNA ends (RACE) procedures to obtain full-length novel peptide precursor nucleic acid sequence data using a SMART-RACE kit (Clontech, Leeds, UK) likewise as per manufacturer’s instructions. Briefly, the 3′-RACE reactions employed a nested universal (NUP) primer (supplied with the kit) and a degenerate sense primer (SP: 5′-GGIATGMGICCICCITGG-3′) (I = deoxyinosine, M = A/C) that was complementary to the signal peptide amino acid sequence. The 3′-RACE reactions were purified and cloned using a pGEM-T vector system (Promega Corporation, Wisconsin, USA) and sequenced using an ABI 3700 automated sequencer (Applied Biosystems, California, USA). The sequence data obtained from the 3′-RACE product was used to design a specific antisense primer (ASP: 5′-CGGCACTATTACTGATAATTGTGCT-3′) to a defined site within the 3′ non-translated region of the novel peptide precursor-encoding transcripts. 5′-RACE was carried out using these primers in conjunction with the NUP primer and the resultant products were purified, cloned and sequenced.

### 2.3. Isolation and Structural Analysis of Novel Peptide

The amino-acid sequence of the LFB was deduced from the cDNA cloning results. To double confirm the amino-acid sequence of the LTB, a second batch of 5 mg lyophilised *L*. *fujianensis* sample was dissolved in 0.5 mL of trifluoroacetic acid (TFA)/water (0.05/99.95; *v*/*v*) and then centrifuged (1100× *g* for 5 min). The supernatant was gradually poured to another tube without disturbing the sediment, and further subjected to reverse phase-high performance liquid chromatography (RP-HPLC) fractionation using a Waters HPLC system installed with an analytical column (Phenomenex C-5; 250 mm × 4.6 mm, California, USA). For further procedures in detail, please kindly refer to previous study [25].

### 2.4. Blast Analysis and Solid-Phase Peptide Synthesis

Once we obtained the open reading frame of LTB, we picked the putative mature peptide sequence of LTB (GLFSVVKGVLKGVGKNVSGSLLDQLKCKISGGC) for BLAST comparison (https://blast.ncbi.nlm.nih.gov/Blast.cgi, BLASTP 2.9.0+, Database nr_v5). The structural similarity of LFB was used for classification into existing peptide families or a structurally-novel peptide family. We also synthesized LFB using an automated PS3 peptide synthesizer (Protein Technologies, Woburn, MA, USA). After synthesis, synthesized replicates of LFB were further purified by RP-HPLC and confirmed its authenticity by MALDI-TOF MS. A sample of this fraction was subjected to MALDI-TOF mass spectrometry using a Perseptive Biosystems Voyager DE mass spectrometer (Applied Biosystems, Warrington, UK) in positive detection mode using α-cyanohydroxycinnamic acid as matrix.

### 2.5. Prediction of Secondary Structures of LFB and Its Physicochemical Properties

LFB’s putative peptide 2D structures were analyzed and predicted by using Garnier-Osguthorpe-Robson (GRO) 2D structure prediction software version 1.1 (http://distill.ucd.ie/distill/). Additional physicochemical properties, such as hydrophilicity, hydrophobicity, helical wheel plots and net charge at neutral pH, were all determined via the Heliquest server menu (http://heliquest.ipmc.cnrs.fr/cgi-bin/ComputParamsV2.py).

### 2.6. Circular Dichroism Spectra of Synthetic Peptide LFB and Detection of Its Antimicrobial Activity Assays

The 2D structure was analyzed and determined by a circular dichroism (CD) spectrometer (Jasco J851, Tokyo, Japan). The analysis method was performed as previous study [26]. The obtained spectra were analyzed with the BeStSel CD online analysis program (test version) for calculating the proportion of LFB’s helical conformation (http://bestsel.elte.hu). The minimal inhibitory concentrations (MICs) for the synthetic peptide LFB were studied using initial qualitative zonal growth inhibition assays against model strains of Gram +ve bacteria *S. aurues* (NCTC 10788), Gram –ve bacteria *E. coli* (NCTC 10418) and yeast *C. albicans* (NCYC 1467). For further procedures in detail, please kindly refer to previous study [27].

### 2.7. Tissue Culture of Maintaining Human Cancer Cell Lines

Human cancer cell lines [NCI-H460 [H460] (ATCC^®^ HTB-177™); MDA-MB-435S (ATCC^®^ HTB-129™); HCT 116 (ATCC^®^ CCL-247™)] and [U-251 MG cell line human (09063001-1VL)] were purchased from the American Type Culture Collection (ATCC) USA and Sigma-Aldrich UK respectively. The cells were cultured using Dulbecco’s Modified Eagle’s Medium (DMEM) medium or RPMI-1640 medium (Invitrogen) depending on different cell lines. In general, the medium was supplemented with 0.1% (*w*/*v*) gentamicin and 10% (*v*/*v*) fetal bovine serum (FBS) (Sigma-Aldrich, Dorset, UK). The cells were then seeded into 150 cm^2^ tissue culture flasks (Nunc, Roskilde, Denmark) for culturing.

### 2.8. Studies on Anti-Proliferative Effects of LFB via MTT Assay and Incucyte Live Cell Imaging Systems

For the MTT assay, 3000 cells/per wells were seeded in the 96 well plates 24 h in advance. Subsequently, fresh medium contains different concentration of LFB (1 pM to 1 μM) was added in to experiment groups, respectively. The fresh medium contained equal amounts of PBS, used as a negative control. At least three replicates were included in each group. Then after 24 h of treatment, we measured the cell viability according to the procedure performed in our previously study [26]. In the IncuCyte assay, ten thousand cancer cells were seeded in six-well plates and treated with or without the peptide LFB (concentration range from the concentration of 100 μM to 1 μM) for 24 h. Then, the IncuCyte ZOOM™ Continuous Live-cell Imaging & Analysis System was used to record cell growth condition for 72 h. The instrument can automatically generate the cell growth curve for every two hours which was calculated by the average cell area attached to the bottom of each well according to the images taken by the instrument. The negative control group and blank control group were treated with equal volume of negative control peptide and PBS, respectively. The alteration of nuclear morphology after LFB treatment was also detected using IncuCyte. As described above, cells were grown overnight in IncuCyte. LFB treated cells were examined by IncuCyte ZOOM™ Continuous Live-cell Imaging & Analysis System. Dead cells were defined on the basis of cellular morphology changes, such as chromatin condensation and/or cell shrinking.

### 2.9. Lactate Dehydrogenase (LDH) Assay

Cells were treated with LFB ranging from 100 μM to 1 μM for 24 h. LDH Cytotoxicity Assay Kit (Cayman Chemical, Ann Arbor, MI, USA) was employed to detect the cytotoxicity of LFB according to the manufacturer’s instructions. For details, please kindly refer to the previous study [27].

### 2.10. Phosphatidylserine Exstrophy Detection (Apoptosis Assay)

Cancer cells were treated with LFB at 100 μM to 1 μM for 2 h. Then, the Alexa Fluor 488 annexin V/Dead Cell Apoptosis Kit (Thermo Fisher Scientific, Waltham, MA, USA) was employed to detect apoptosis according to the manufacturer’s instructions. Flow cytometer BD Accuri™ C6 was used for the analysis and blank control group was treated with equal volume of PBS.

### 2.11. Haemolysis Activity Study

A 2% (*v*/*v*) suspension of red blood cells was prepared from defibrinated horse blood (TCS Biosciences Ltd., Claydon, UK). Red blood cell suspension samples (200 μL) were incubated with a range of LFB concentrations (1–512 mg/L) for 60 min at 37 °C. Lysis of red cells was assessed by measurement of optical density at *λ* = 550 nm using an ELISA plate reader (BioliseBioTek EL808, Winooski, USA). Negative controls employed consisted of a 2% (*v*/*v*) red cell suspension and sodium PBS in equal volumes; and positive controls consisted of a 2% (*v*/*v*) red cell suspension and an equal volume of PBS containing 2% (*v*/*v*) of the non-ionic detergent, Triton X-100 (Sigma-Aldrich, Dorset, UK).

### 2.12. Statistical Analysis

Statistical analysis was performed using Version 6.0 software of GraphPad Prism. The *p*-values were calculated by Student’s *t*-test from the mean values of the indicated data. Significant differences were demonstrated with asterisks (* *p* < 0.05; ** *p* < 0.01; *** *p* < 0.001).

## 3. Results

### 3.1. Isolation and Structural Characterization of the Novel Antimicrobial Peptide

**Figure 1:**
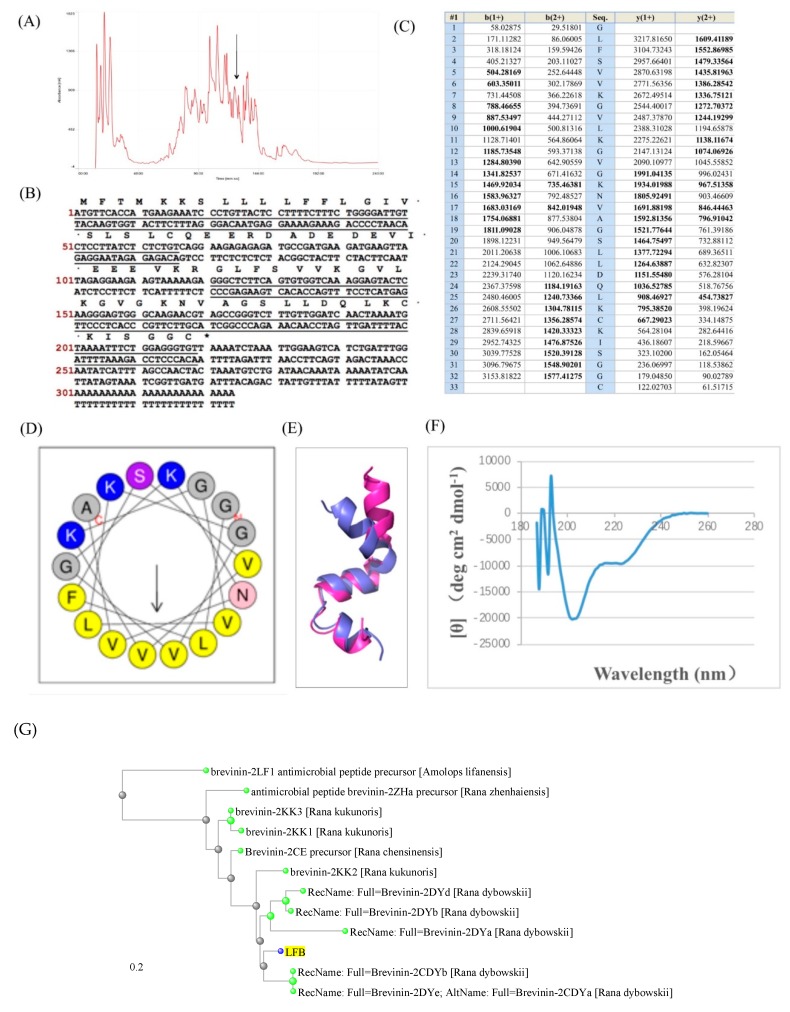
(**A**) Region of reverse phase high performance liquid chromatography (HPLC) chromatogram of *Limnonectes fujianensis* skin secretion indicating the elution position/retention time (arrow) of absorbance peak containing the peptide, *Limnonectes fujianensis* Brevinvin (LFB). (**B**) Nucleotide and translated open-reading frame amino acid sequence of the cloned cDNA encoding the biosynthetic precursor of LFB. The putative signal peptide is double-underlined and the mature LFB sequence is single-underlined. The stop codon was indicated with an asterisk. (**C**) Expected singly- and doubly-charged *b*-ion and *y*-ions arising from fragmentation of LFB as predicted using the MS-Product program available through Protein Prospector Online (http://prospector.ucsf.edu/prospector/mshome.htm). Observed fragment ions are indicated in bold typeface and are underlined. (**D**) Domain architecture of pre-LFB. Residues 1–22 constitute the putative signal peptide. Residues 23–40 constitute the acidic spacer peptide region typified by classical -KR- (-Lys-Arg-) pro-peptide convertase processing sites (italicized and in bold typeface). The single copy of mature LFB (residues 41–73) is underlined and in bold typeface. (**E**) Predicted protein structure of LFB. (**F**) Circular dichroism (CD) spectra of LFB. The spectra have subtracted buffer. (**G**) The distance tree of LFB using NCBI BLAST.

The lyophilized skin secretion of *L*. *fujianensis* was subjected to RP-HPLC and the resultant chromatogram is shown in Figure 1A. The computed molecular mass of this novel LFB was 3276 Da. The interrogation of HPLC fractions using MALDI-TOF MS resulted in the identification of the elution position of LFB. Primary structural analysis was performed by MS/MS fragmentation sequencing using the electrospray MS and the result is summarized in Figure 1B,C. The two-pronged approach was applied to obtain the amino-acid sequence of LFB. We deduced the putative mature peptide sequence and molecular weight of LFB by enzyme cutting sites and stop codon. In the meanwhile, we used RP-HPLC/MS to detect the molecular weight and amino-acid sequence of every individual peptide in the frog skin secretion peptide pool. Then we compared these two results, if the deduced molecular weight of LFB was matched in the RP-HPLC/MS results. We take the amino-acid sequence as the sequence of LFB.

### 3.2. Molecular Cloning of the Novel Antimicrobial Peptide from the Skin Cdna Library of Limnonectes fujianensis

A single cDNA encoding the precursor of the novel peptide was consistently and repeatedly cloned from the library using the protocol described. The nucleotide and translated open reading frame amino acid sequences and the domain architectures were shown in Figure 1B,D,E. Basically, the precursor consists of a putative signal peptide region of 22 amino acid residues, an acidic spacer domain, a typical –KR– pro-peptide convertase processing site and mature active peptide-encoding domain of 33 amino acid residues.

### 3.3. Secondary Structures Prediction of Putative Peptide LFB

The helical wheel plots and secondary structures of LFB were predicted with online analysis tools. It also had a hydrophobic face consisting of V, L, V, V, V, L, F, which were illustrated in Figure 1D, and the secondary structure was determined by a CD spectrometer (Figure 1F). These plots revealed and amphipathic character in the peptide with hydrophobic amino acid residue (V, L, F) sidechains essentially occurring on one face of the molecule and hydrophilic amino acid residue (K, S) sidechains occurring on the other. The distance tree result using BLAST indicated that our novel antimicrobial peptide LFB showed high structural similarity with Brevenins. Thus, we detected the antimicrobial activity of LFB.

### 3.4. Antimicrobial Activities of Synthetic LFB

**Figure 2:**
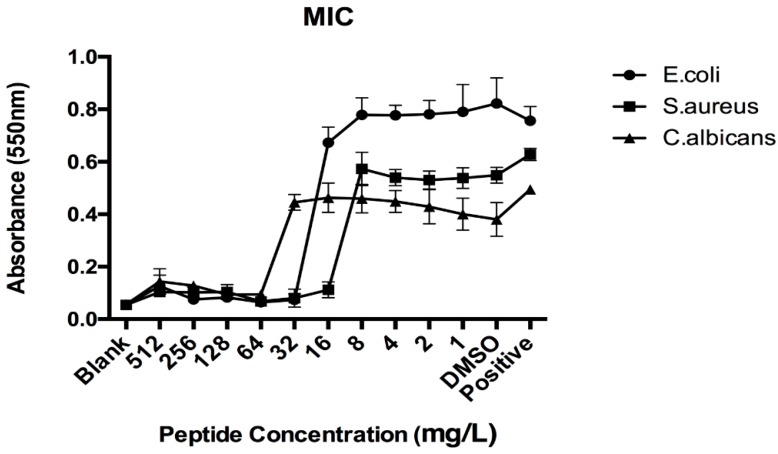
Minimal inhibitory concentration (MIC) curves obtained following incubation of synthetic LFB with *Escherichia coli* (NCTC 10418), *Staphylococcus aureus* (NCTC 10788) and *Candida albicans* (NCPF 1467).

Synthetic LFB exhibited growth inhibitory activity against the Gram-positive bacterium *S*. *aureus* at concentrations at and above 16 mg/L, against the Gram-negative bacterium *E*. *coli* at concentrations at or above 32 mg/L and against the potentially-pathogenic yeast, *C*. *albicans,* at concentrations at or above 64 mg/L (Figure 2). Each assay was carried out over three individual experiments and the standard errors were typically <5% of the mean.

### 3.5. Anti-Proliferative Effects of LFB on Human Cancer Cells

**Figure 3:**
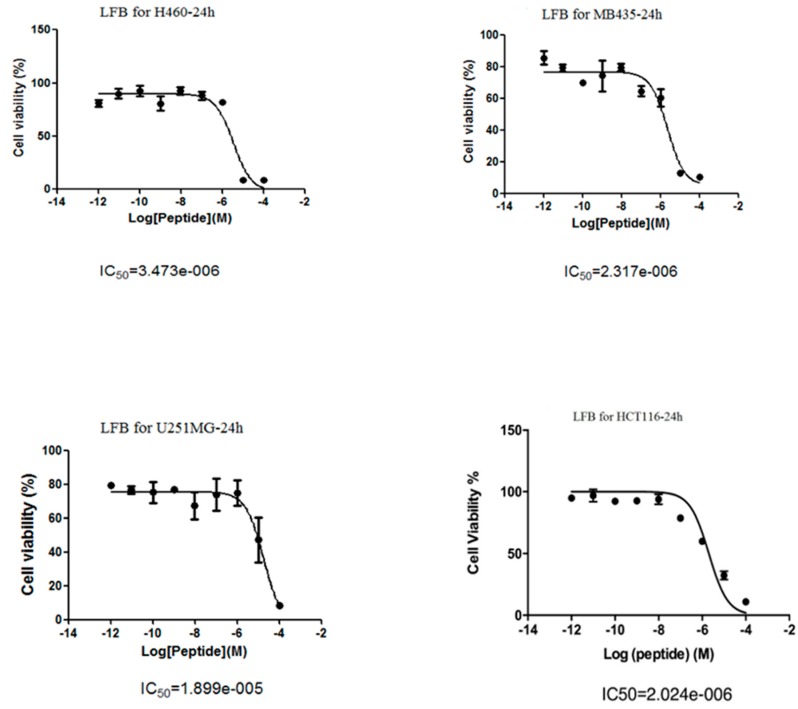
Anti-proliferative effects of LFB on a panel of human cancer cell lines after 24 h incubation. Panels illustrate the effect on NCI-H460 (human non-small cell lung cancer cell line), H838MDA-MB-435S (human breast cancer cell line), U251MG (human neuronal glioblastoma cell line), HCT116 (human colon cancer cell) respectively. IC_50_ values were given for each peptide in panels below graphs.

The data obtained for LFB in this assay was graphically represented. LFB was only active in four human cancer cell lines (H460, MB435, U251MG and HCT116) and the IC_50_ values were 3.47 μM, 18.99 μM, 2.32 μM and 2.02 μM, respectively. LFB was found to have the best anti-cancer activity on human colon cancer cell HCT116. As a result, HCT116 was chosen as a lead cell line for further study. The relationships between cell viabilities and peptide concentrations are shown in Figure 3.

### 3.6. Proliferation Curve Generated Through Incucyte Live Cell Imaging System

**Figure 4:**
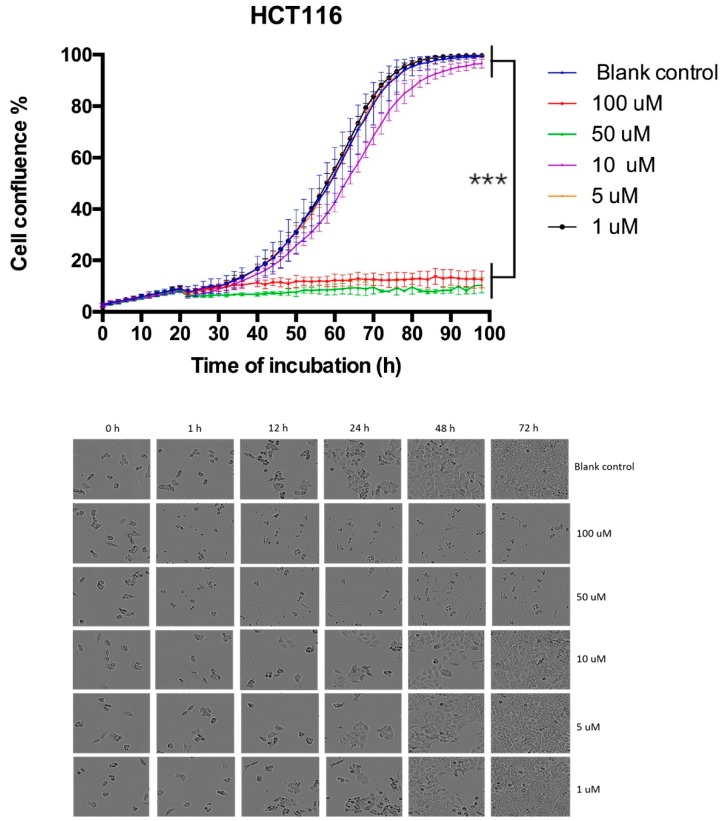
Growth curve of HCT116 treated with gradient concentrations of the peptide LFB. Note that proliferation of HCT116 cells were inhibited in a dose-dependent manner with peptide treatment, and the blank control group was treated with equal amounts of PBS. The visualized results were randomly picked from 25 pictures, which were caught continually for every well. The levels of significance are * *p* < 0.05; ** *p* < 0.01; *** *p* < 0.001; **** *p* < 0.001.

Cancer cell HCT116 was treated with LFB and incubated in the IncuCyte live cell imaging system. As shown in Figure 4, the proliferation rate of HCT116 treated with LFB was much slower than the blank control group. This promising result demonstrated that LFB could inhibit cancer cell proliferation in a dose-dependent manner.

### 3.7. Apoptosis Assay (Phosphatidylserine Exstrophy Detection)

**Figure 5:**
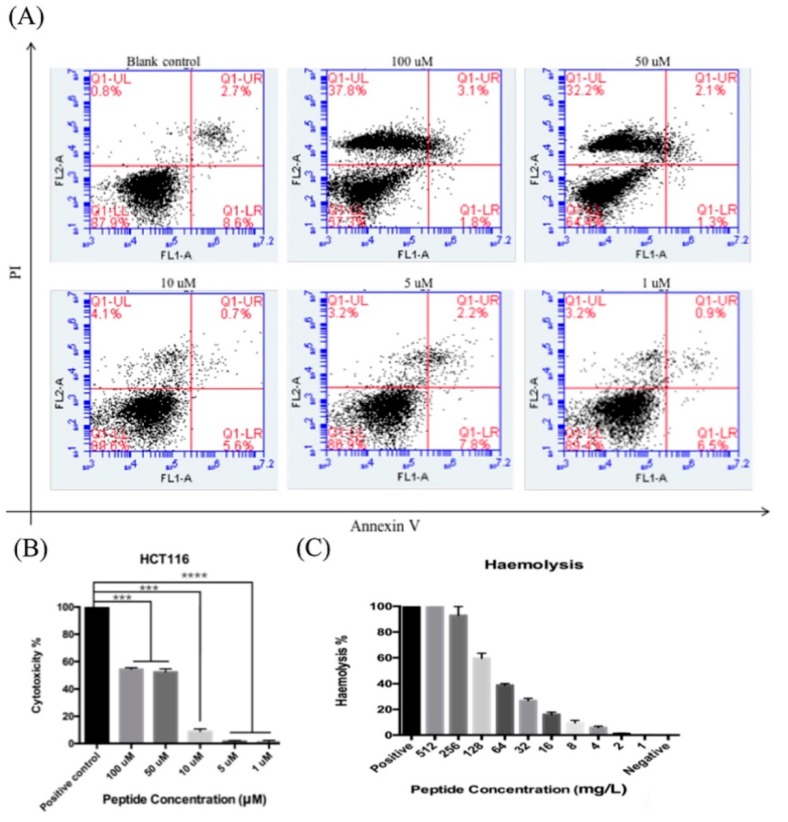
(**A**) The apoptotic and cell toxicity of LFB (**B**) Cytotoxicity of LFB on cancer cell HCT116. Cytotoxicity was calculated based on LDH release. The levels of significance are * *p* < 0.05; ** *p* < 0.01; *** *p* < 0.001; **** *p* < 0.001. (**C**) The hemolytic activity of synthetic LFB.

Annexin V and Propidium Iodide (PI) staining along with flow cytometer analysis were employed in order to determine if LFB inhibits cancer cells via cell apoptosis or by other modes of actions. The readouts from flow cytometry divided the cells into four groups: normal (Annexin V^−^/PI^−^), early apoptosis (Annexin V^+^/PI^−^), late apoptosis (Annexin V^−^/PI^+^) and dead (Annexin V^+^/PI^+^). From the results in Figure 5A, LFB didn’t induce apoptosis in HCT116, directly causing cell death when at high concentrations when compared to the blank control group.

### 3.8. LDH Assay of Synthetic Peptide LFB

To further verify that LFB would induce cancer cell death after treatment, an LDH assay was employed. The cytotoxicity of LFB on HCT116 was shown in Figure 5B. From the results, LFB increased LDH release in cancer cells, with LDH amounts all above 80% (the positive control was regarded as 100%), which indicate that LFB possibility affects cancer cell proliferation through disrupting its cell membrane.

### 3.9. Hemolysis Assay of Synthetic Peptide LFB

This assay demonstrated that LFB exhibited significantly high hemolytic activity at a concentration of 16 mg/L and above (Figure 5C). Each assay was carried out over three individual experiments and the standard errors were typically <5% of the mean.

## 4. Discussion

In the last two decades, the development of microbial resistance has resulted in many problems internationally for the treatment of infectious diseases of both humans and their livestock [28,29,30,31]. This acquired resistance poses a major threat to public health and food supplies. It is imperative that both academic and industrial bio-scientists are united in the search for some novel molecular solutions to this rapidly growing worldwide problem [32]. AMPs are now well-recognized to constitute a fundamental component of innate immunity and molecular defense in many living organisms, including bacteria [33,34,35,36]. AMPs demonstrated a certain characteristic to be potent and also broad spectrum in their action which can directly kill both Gram −ve and Gram +ve bacteria, including those strains which are resistant to traditional forms of antibiotics. Secretions from amphibian skin have been shown to have huge amount of natural source and diversity of antimicrobial peptides [37]. These peptides have demonstrated to have good efficacy against several Gram −ve and Gram +ve bacteria, fungi, protozoans and certain viruses (e.g., HIV). In the last two decades, AMPs are thought to be particularly abundant in skin secretion of frogs, where they serve as a defense against surface microbial colonization, a consequence of both life forms being particularly abundant in the same biotopes [38].

According to the studies in the last two decades, there are many different antibacterial mechanisms have been reported. However, the most well recorded one is classified as a non-specific interaction with the membrane with different models, such as the Barrel–Stave model, Carpet model, and Toroidal model [39]. In one of the recent cancer studies, Brevinin showed that it preferentially interacts with different types of cancer cells because the outer membrane surface of these cells has an additional −ve charge due to the presence of higher levels/numbers of negatively charged phosphatidylserines, O-glycosylated mucines and/or microvilli, which leads to increases in the overall surface area of the membrane [40]. Compared to other kinds of Brevinins reported, LFB showed the most potent anticancer and antimicrobial activities, like Brevinin 1GHa (FLGAVLPVAGKLVPAAICKISKKC) and its subfamily 1GHb, 1GHc, and also the Brevinin 1E, 1HSa, 1WY7, 1JDc, 1PTc, 1LTd. Most of these reported Brevinins showed less potent or no anticancer activities, with some of them even having longer sequences, so LFB has more potential as a drug candidate [41]. Unfortunately, most of the Brevinins, including LFB which we identified in this study, have the strong hemolytic properties that impede their application to being developed as antimicrobial drug candidates. However, some recent experiments indicate that this hemolytic ‘side effect’ could be decreased by further structural modifications. For example, in Brevinin-1E (FLPLLAGLAANFLPKIFCKITRKC) which was identified from the skin secretion of *Rana esculenta*, if the C-terminal sequence CKITRKC was removed and re-positioned at its central position (FLPLLAGLCKITRKCAANFLPKIF), this structural modification could lead to a considerable reduction of its hemolytic activity without loss of any antibacterial activity [42,43,44,45]. Therefore, the structural diversity analysis within discrete families of natural bioactive functional peptides, especially for those which are homologues of endogenous mammalian regulatory peptides, could provide libraries of specific/unique antagonists and agonists for exploring the structure/activity requirements of interactions with endogenous molecular targets as a solid support and evidence to rational analogue design [45]. These approaches have done much to educate pharmaceutical bioengineers about peptide-based prototype drug design, which serve as examples of suggestions and shed a light to open up an opportunity for further studies on both peptides as leads for both therapeutic applications [46].

In summary, a newly reported bioactive peptide LFB was identified from the frog skin secretion of *L. fujianensis* for the first time. LFB demonstrated broad spectrum and highly potent antimicrobial activity against Gram +ve bacteria, Gram −ve bacteria, and fungi. Furthermore, LFB also showed the ability to inhibit the growth of several types of cancer cell lines. With the purpose of developing LFB as a new antimicrobial prototype drug, structural-functional modification should be seriously taken into consideration for reducing its hemolytic activity. In general, the current study suggests that LFB is a promising drug candidate for the development of novel antibiotic class, and these findings may pave a new insight into natural antimicrobial prototype drug design.
